# Raman Spectroscopy Can Distinguish Glyphosate-Susceptible and -Resistant Palmer Amaranth (*Amaranthus palmeri*)

**DOI:** 10.3389/fpls.2021.657963

**Published:** 2021-06-04

**Authors:** Vijay Singh, Tianyi Dou, Mark Krimmer, Shilpa Singh, Dillon Humpal, William Z. Payne, Lee Sanchez, Dmitri V. Voronine, Andrey Prosvirin, Marlan Scully, Dmitry Kurouski, Muthukumar Bagavathiannan

**Affiliations:** ^1^Department of Soil and Crop Sciences, Texas A&M University, College Station, TX, United States; ^2^Department of Biochemistry and Biophysics, Texas A&M University, College Station, TX, United States; ^3^Department of Physics and Astronomy, Texas A&M University, College Station, TX, United States

**Keywords:** herbicide resistance diagnostics, plant stress, field scouting, precision weed management, remote sensing, vibrational spectrum

## Abstract

The non-judicious use of herbicides has led to a widespread evolution of herbicide resistance in various weed species including Palmer amaranth, one of the most aggressive and troublesome weeds in the United States. Early detection of herbicide resistance in weed populations may help growers devise alternative management strategies before resistance spreads throughout the field. In this study, Raman spectroscopy was utilized as a rapid, non-destructive diagnostic tool to distinguish between three different glyphosate-resistant and four -susceptible Palmer amaranth populations. The glyphosate-resistant populations used in this study were 11-, 32-, and 36-fold more resistant compared to the susceptible standard. The 5-enolpyruvylshikimate-3-phosphate synthase (*EPSPS*) gene copy number for these resistant populations ranged from 86 to 116. We found that Raman spectroscopy could be used to differentiate herbicide-treated and non-treated susceptible populations based on changes in the intensity of vibrational bands at 1156, 1186, and 1525 cm^–1^ that originate from carotenoids. The partial least squares discriminant analysis (PLS-DA) model indicated that within 1 day of glyphosate treatment (D1), the average accuracy of detecting herbicide-treated and non-treated susceptible populations was 90 and 73.3%, respectively. We also found that glyphosate-resistant and -susceptible populations of Palmer amaranth can be easily detected with an accuracy of 84.7 and 71.9%, respectively, as early as D1. There were relative differences in the concentration of carotenoids in plants with different resistance levels, but these changes were not significant. The results of the study illustrate the utility of Raman spectra for evaluation of herbicide resistance and stress response in plants under field conditions.

## Introduction

Weeds compete with crop plants for critical resources and cause severe yield losses, if not managed adequately (Oerke, 2006). Herbicides are the most commonly used tool for weed control in modern agriculture; however, repeated use of few herbicide modes of action (MOA) has led to a widespread evolution of herbicide-resistant weeds in cropping systems. Currently, 263 weed species have evolved resistance to 167 herbicides used globally (Heap, 2020). In the United States cropping systems, weeds resistant to glyphosate (the active ingredient in the herbicide Roundup^®^) have become a major production challenge (Garetson et al., 2019). Glyphosate inhibits the 5-enolpyruvylshikimate-3-phosphate synthase (EPSPS) enzyme in plants and leads to the depletion of the aromatic amino acids tryptophan, tyrosine, and phenylalanine. The first case of glyphosate resistance in Palmer amaranth (*Amaranthus palmeri* S. Wats.), the most troublesome weed in the United States cropping systems (Van Wychen, 2017), was confirmed in 2004 in Georgia (Culpepper et al., 2006). Currently, 30 states in the United States have reported the occurrence of glyphosate-resistant Palmer amaranth (Heap, 2020).

Glyphosate-resistant weeds have been managed with alternative herbicides of different MOA. However, early detection of resistance is critical in order to implement measures in a timely manner. The common approach of confirming resistance by collecting mature seeds from putative resistant plants and then testing the seedlings with the herbicide is time consuming (Beckie et al., 2000), and by the time the diagnosis results are available, it is often too late to implement effective field management strategies. Sometimes, farmers attempt to apply a different herbicide immediately after observing weed control failure. However, glyphosate applications require a waiting period of about 10–14 days before plant response can be determined; given the rapid growth rate of Palmer amaranth (Horak and Loughin, 2000), the resistant plants can grow to large sizes before subsequent herbicide applications can be made, rendering such applications largely ineffective. There is a critical need for novel technologies that can facilitate early stage detection of herbicide resistance in weed populations.

Raman spectroscopy (RS) has emerged as an analytical tool for rapid and non-destructive diagnostics of abiotic (Altangerel et al., 2017) and biotic stresses in plants (Egging et al., 2018; Farber and Kurouski, 2018; Farber et al., 2019b; Sanchez et al., 2019a, b, c). RS measures the vibrational spectrum of the analyzed sample that allows for determining its structure (Thomas, 1999; Petry et al., 2003). Thus, RS is considered as a molecular “fingerprinting” technique, owing to its ability to identify substances and their chemical compositions (McCreery, 2005; Schulz and Baranska, 2007). In plants, Raman spectra contain vibrational bands that can be assigned to carbohydrates, carotenoids, proteins, and phenylpropanoids (Sene et al., 1994; Schulz and Baranska, 2007). Measurements using the traditional confocal RS units are carried out under controlled conditions. However, the improvements with handheld Raman spectrometers (Hager et al., 2018; Sanchez et al., 2020a) over the past decade have allowed for on-site diagnosis of biotic and abiotic stresses in plants (Farber et al., 2019a). For instance, fungal diseases in corn can be detected with 100% accuracy using RS (Farber and Kurouski, 2018). It has been demonstrated that RS is capable of diagnosing ergot, black tip, and mold on wheat and sorghum (Egging et al., 2018). Additionally, RS has been shown effective in detecting the presence of insects inside intact cowpea seeds with high statistical accuracy (Sanchez et al., 2019a). RS could be used to distinguish between healthy, Huanglongbing (early and late stage)-infected citrus trees and those suffering from nutrient deficiencies (Sanchez et al., 2019b, c). Based on this knowledge, we hypothesized that RS can be used for detection of herbicide resistance in weeds. In the present study, we investigated the accuracy of RS in differentiating between several different glyphosate-susceptible and -resistant Palmer amaranth populations.

## Materials and Methods

### Plant Material Characterization

Three previously confirmed glyphosate-resistant (TX15-10, TX15-12-2, TX15-14-1) and four glyphosate-susceptible (TX15-2, TX15-13-2, TX15-29, TX16-10) populations were used (Garetson et al., 2019). Although these populations were known to be resistant or susceptible to glyphosate, the extent of sensitivity to this herbicide as well as the mechanism of resistance (*EPSPS* gene copy numbers) is yet to be determined, which will help make a more informed interpretation of the findings. For this purpose, a herbicide dose–response assay and a gene copy number analysis were conducted.

#### Glyphosate Dose–Response

**FIGURE 1:**
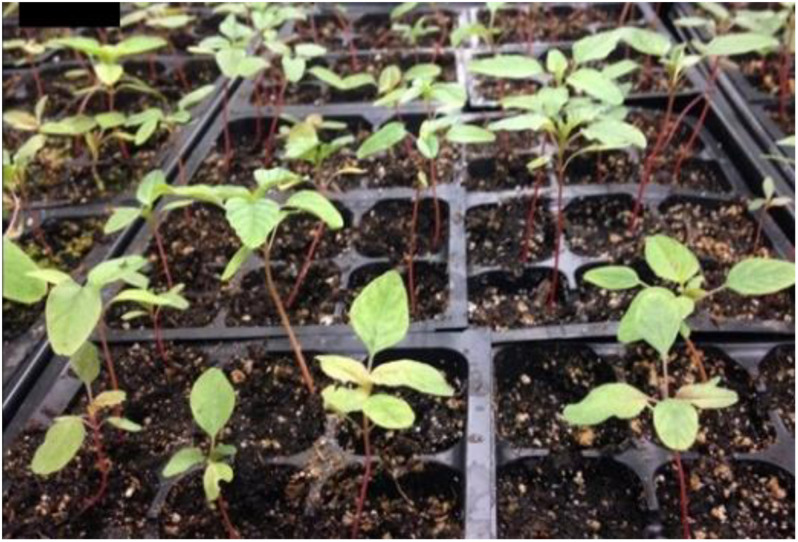
Growth stage (8- to 10-cm seedlings) of the Palmer amaranth plants when treated with glyphosate (1 × = 868 g ae ha^–1^). An 830-nm continuous-wave (CW) laser was targeted on the leaf blade, and the leaf area near the veins was avoided for uniformity.

Seeds of glyphosate-resistant and -susceptible Palmer amaranth populations were planted in six-cell trays (Figure 1) filled with potting soil mix (LC1 Potting Mix, Sun Gro Horticulture Inc., Agawam, MA, United States) in a greenhouse at Texas A&M University, College Station, TX, United States, during Fall 2019. Resistant and susceptible populations were treated with eight different doses of glyphosate: 0, 217, 434, 868, 1736, 3472, 6944, and 13888 g ae ha^–1^ for the resistant population and 0, 54.3, 108.5, 217, 434, 868, 1736, and 3472 g ae ha^–1^ for the susceptible population. Glyphosate was applied at 1× the recommended label rate (868 g ae ha^–1^) at the 8–10-cm-tall seedling stage (Figure 1), using a spray chamber fitted with a flat fan nozzle (TeeJet XR110015) calibrated to deliver 140 L ha^–1^ of spray volume, operating at 4.8 kmph. The greenhouse was maintained at a day/night temperature regime of 30/26°C and a photoperiod of 14 h. The experiment was conducted in a randomized complete block design with four replications. Percent survival and injury were evaluated at 21 days after treatment (DAT). Data were analyzed using SigmaPlot v.13 (Systat Software, Inc., San Jose, CA, United States). A three-parameter logistic regression equation (1) provided the best fit for the herbicide injury data.

(1) Y=c/[1+e]{-a(x-b)}

where *Y* is the injury (%), *a* is the slope of the curve, *b* is the inflection point, *c* is the lower asymptote, and *x* is the herbicide dose.

The regression equations were used to calculate the amount of herbicide that caused 50% injury/growth reduction (*GR*_50_). The *GR*_50_ value of the resistant population divided by the averaged *GR*_50_ of the susceptible standard provided the resistance ratio (R/S) values for each resistant population.

#### *EPSPS* Gene Copy Number

The leaf samples of non-treated susceptible populations and confirmed resistant populations (treated) were collected at 21 DAT. DNA was extracted from leaf tissues, according to the protocol provided by Takara DNA isolation kit (Takara Bio Inc., Mountain View, CA, United States, Cat # 9194), with the exception that the dry pellets were resuspended in 20 μl distilled, deionized water instead of the TE buffer. The DNA concentration was quantified using NanoDrop 2000 (Thermo Fisher Scientific, Wilmington, DE, United States), and DNA was diluted according to the requirement. The *EPSPS* gene copy number in each of the populations was determined in comparison to the acetolactate synthase (*ALS*) gene (a positive control) using a qPCR. The forward and reverse primers used for the *EPSPS* and *ALS* genes are as follows: EPSF1 (5′ATGTTGGACGCTCTCAGAACTCTTGGT3′) × EPSR8 (5′TGAATTTCCTCCAGCAACGGCAA3′) and ALSF2 (5′GCTGCTGAAGGCTACGCT3′) × ALSR2 (5′GCG GGACTGAGTCAAGAAGTG3′) (Gaines et al., 2010). A 25-μl reaction mix was prepared using SYBR Green Supermix (12.5 μl) (Bio-Rad, Hercules, CA, United States), forward and reverse primers (10 μM), and gDNA (1 ng). The qPCR was run at 95°C for 15 min followed by 40 cycles of 95°C for 30 s and 60°C for 60 s. A negative control (no DNA template) was also used. Data were analyzed using a method to calculate genomic copy number of *EPSPS* relative to *ALS*, as ΔCt = (Ct *ALS* - Ct *EPSPS*). An increase in genomic *EPSPS* copy number was expressed as 2^ΔCt^ (Gaines et al., 2011).

### Raman Spectroscopy

**FIGURE 2:**
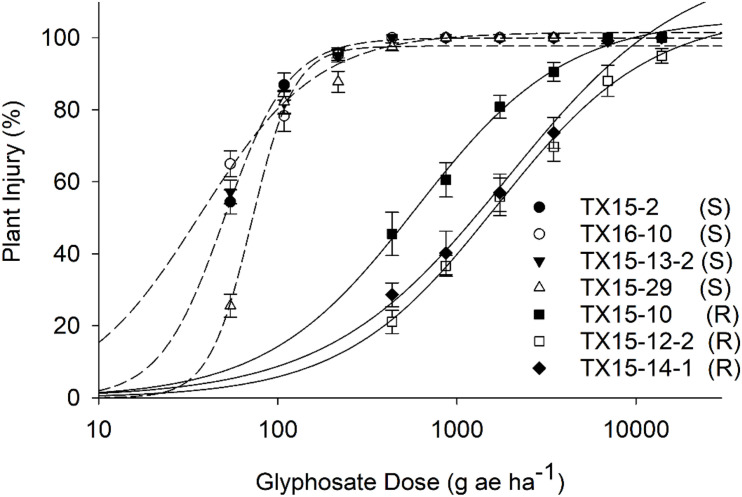
Dose–response to glyphosate of the resistant (R) and susceptible (S) populations based on injury ratings (%) at 21 days after treatment. Injury data were recorded on a scale of 0–100% (0% = no injury and 100% = plant death). Dose–response curves were plotted on mean values of plant injuries (%) and error bars represent standard error (SE) of means.

Raman spectra were determined with a handheld Resolve Agilent spectrometer (Agilent, Santa Clara, CA, United States) equipped with an 830-nm laser source. The spectra were collected with 1-s acquisition time and 495-mW power. Four spectra were collected from each leaf from four quadrants on the adaxial side of the leaf of each resistant and susceptible plant before herbicide treatment (D0) and after 1 day (D1) and 2 days (D2) of herbicide application. For each of the treatment groups (resistant and susceptible) at each time point, 30 spectra were collected, resulting in a total of 1584 spectra. Following this, the spectra were baselined using the handheld instrument software. Initially, spectra were collected from one herbicide-resistant (TX15-14-1) and one susceptible (TX15-13-2) population only for standardization of the instrument. Later, about 600 Raman spectra from leaves of three herbicide-resistant (TX15-14-1, TX15-10, and TX15-12-2) and four herbicide-susceptible (TX16-10, TX15-2, TX15-29, and TX-13-2) populations (Figure 2) were collected and normalized to the 1438-cm^–1^ band, representing CH_2_ and CH_3_ vibration (Table 1; Farber et al., 2019b). This chemical group is present in nearly all biological molecules, which makes the normalization unbiased to any specific chemical component of the weed leaf.

**TABLE 1:** Vibrational band assignments for Palmer amaranth leaf spectra.

Band	Vibration mode	Assignment
747	γ(C–O–H) of COOH	Pectin (Farber et al., 2019b)
915	*ν*(C–O–C) in plane, symmetric	Cellulose, phenylpropanoids (Farber et al., 2019b)
1001	In-plane CH_3_ rocking	Carotenoid (Farber et al., 2019b)
1047	*ν*(C–O) + *ν*(C–C) + δ(C–O–H)	Cellulose, phenylpropanoids (Farber et al., 2019b)
1085	*ν*(C–O–H) next to aromatic ring + δ(CH)	Carbohydrate (Sanchez et al., 2020b)
1156	C–C stretching; *ν*(C–O–C), *ν*(C–C) in glycosidic linkages, asymmetric ring breathing	Carotenoid (Farber et al., 2019b)
1186	*ν*(C–O–H) next to aromatic ring + δ(CH)	Carotenoids (Ruban et al., 2001)
1213	δ(C–C–H)	Carotenoids (Ruban et al., 2001)
1268	Guaiacyl ring breathing, C–O stretching (aromatic)	Phenylpropanoids (Farber et al., 2019b)
1285	δ(C–C–H)	Aliphatics (Farber et al., 2019b)
1326	δCH_2_ bending	Aliphatics, cellulose, lignin (Farber et al., 2019b)
1387	δCH_2_ bending	Aliphatics (Farber et al., 2019b)
1438	δ(CH_2_) + δ(CH_3_)	Aliphatics (Farber et al., 2019b)
1525	−C = C-(in plane)	Carotenoid (Farber et al., 2019b)
1607	*ν*(C–C) aromatic ring + δ(CH)	Phenylpropanoids (Farber et al., 2019b)
1690	*ν*(C = O)	Carboxyl groups (Sanchez et al., 2020b)

### Statistical Analysis

The Raman spectra data were imported into the MATLAB R2019a add-on PLS_Toolbox 8.6.2 (Eigenvector Research Inc.) for statistical analyses. The preprocessing of the spectra included mean centering and area normalization for partial least square discriminant analysis (PLS-DA), which is a suitable analysis method for spectral data (Lee et al., 2018). Analysis of variance (ANOVA) was also performed in MATLAB R2019a add-on PLS_Toolbox 8.6.2 to compare carotenoid bands (at 1186 cm^–1^ and 1213 cm^–1^) between the herbicide-resistant and -susceptible plants after treating with glyphosate. Matthew’s correlation coefficient is generated by MATLAB R2019a add-on PLS_Toolbox 8.6.2 (Eigenvector Research Inc.) for the binary classification model. The score of MCC is based on four confusion matrix categories: true positive, false negative, true negative, and false positive to reflect the quality of prediction model (Chicco and Jurman, 2020).

## Results and Discussion

### Herbicide Resistance Characterization

The estimated *GR*_50_ values for TX15-10, TX15-12-2, and TX15-14-1 were 537, 1780, and 1568 g ae ha^–1^, respectively (Table 2 and Figure 2). The resistance ratios have indicated that the resistant populations TX15-10, TX15-12-2, and TX15-14 were 11-, 36-, and 32-fold more resistant to glyphosate, respectively, compared to the susceptible standard (average *GR*_50_ = 40.3) (Table 2 and Figure 2). The variation (low and high) in resistance levels of the tested resistant populations in the current study presented an appropriate case for differentiation through RS.

**TABLE 2:** *GR*_50_^a^ values and resistance levels to glyphosate in the Palmer amaranth (*Amaranthus palmeri*) populations used in the study.

Population^b^	RMSE	*R*^2^	*GR*_50_ (g ae ha^–1^)	R/S^c^
TX15-10 (R)	15.05	0.65	537	11.0
TX15-12-2 (R)	16.43	0.71	1780	36.3
TX15-14-1 (R)	16.82	0.72	1578	32.2
TX15-2 (S)	7.08	0.80	47	–
TX16-10 (S)	8.76	0.86	35	–
TX15-13-2 (S)	6.53	0.79	39	–
TX16-10 (S)	9.41	0.87	75	–

*^a^GR_50_ is the herbicide concentration that reduced plant growth by 50% based on visible injury measured at 21 days after treatment with glyphosate. ^b^Populations denoted with R in parenthesis indicate glyphosate-resistant populations and those with S indicate susceptible populations. ^c^R/S ratio was obtained by dividing the GR_50_ of the R population by the average GR_50_ of the four S populations.*

Small spectroscopic changes can be used for confirmatory differentiation of glyphosate-resistant and -susceptible plant populations. The PLS-DA analysis of Raman spectra indicates that glyphosate-treated and non-treated susceptible populations can be differentiated with an average accuracy of 93.45% and 82.1% at D1 and D2, respectively (Table 3). This is a confirmatory evidence that Raman system can detect small variations between healthy and glyphosate-stressed plants within 1 or 2 days of herbicide application.

**TABLE 3:** PLS-DA confusion matrix for treated and non-treated susceptible population TX15-2.

	Members	True prediction rate (TPR%)	Predicted as non-treated	Predicted as treated
One day after treatment (D1)				
Non-treated	30	90	27	3
Treated	15	73.3	4	11
Matthew’s correlation coefficient (D1) = 0.640				
Two days after treatment (D2)				
Non-treated	32	96.9	31	1
Treated	11	90.9	1	10
Matthew’s correlation coefficient (D2)^a^ = 0.878				

*^a^Matthew’s correlation coefficient is generated by MATLAB R2019a add-on PLS_Toolbox 8.6.2 (Eigenvector Research Inc.) for the binary classification model. The score of MCC is based on four confusion matrix categories: true positive, false negative, true negative, and false positive to reflect the quality of prediction model.*

### *EPSPS* Gene Copy Number

**FIGURE 3:**
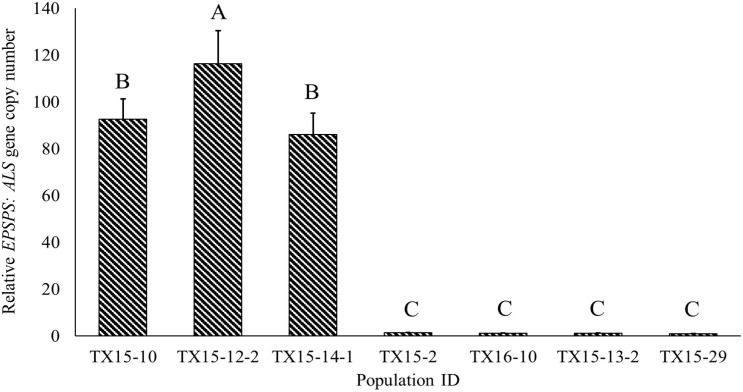
Variability in relative *EPSPS:ALS* gene copy numbers among the Palmer amaranth populations resistant (TX15-10, TX15-12-2, and TX15-14-1) or susceptible (TX15-2, TX15-13-2, TX15-29, and TX16-10) to glyphosate. Each population had four biological replicates (samples) and three technical replicates (*n* = 12). These susceptible populations were known standards, and resistant populations were selected based on the previous study (Garetson et al., 2019). Data were plotted on mean values and error bars indicate standard error (SE) of means. Means represented with different letters are significantly different (Tukey’s honest significance test; HSD, α = 0.05).

The susceptible *A. palmeri* populations used in the current study had a single copy of *EPSPS* (Figure 3). However, relative gene copy numbers in the resistant populations (TX15-10, TX15-12-2, and TX15-14-1) ranged from 86 to 116. In general, *EPSPS* gene amplification is a common resistance mechanism among glyphosate-resistant *Amaranthus* spp. (Singh et al., 2018), which results in increased *EPSPS* enzyme production, allowing the resistant populations to overcome the effect of glyphosate (Gaines et al., 2010). The difference in the number of *EPSPS* copies detected among the resistant populations used in the current study corroborates with the variation in sensitivity of these populations to glyphosate (11- to 36-fold; Table 2). The variation in *EPSPS* gene expression may lead to differential physiological response in Palmer amaranth populations, which may in turn influence Raman spectra.

### Raman Spectra

**FIGURE 4:**
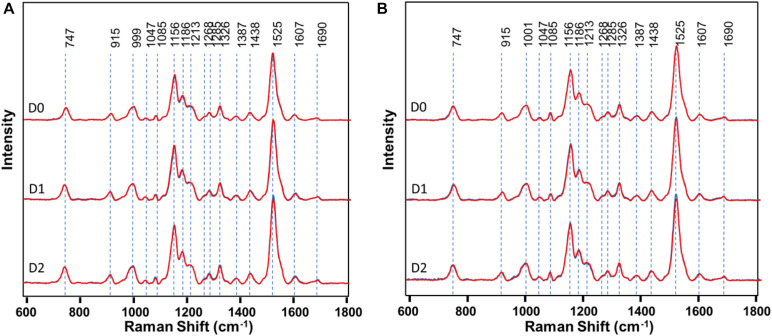
Raman spectra of **(A)** resistant (TX15-14-1) and **(B)** susceptible (TX15-2) populations of Palmer amaranth collected at D0 (non-treated), D1 and D2. Here, D1 and D2 indicate observations conducted at 1 and 2 days after herbicide treatment, respectively. Raman spectra collected from leaves of plants with no herbicide applied (blue curves) were measured at the same time points with those that were sprayed with the herbicide (red). Most of the spectra with no herbicide (blue) was masked by that of herbicide treated (red). The Raman spectra were normalized to total spectral area.

First, RS was used to determine structural changes in a single herbicide-resistant (TX15-14-1) and -susceptible (TX15-2) Palmer amaranth population before (D0) and after (D1 and D2) glyphosate application (Figure 4).

Raman spectra of Palmer amaranth leaves exhibited vibrational bands originating from cellulose, phenylpropanoids, pectin, proteins, and carotenoids (Figure 4 and Table 1). We also observed bands that correspond to the CH_2_ and CH_3_ vibrations that could be assigned to aliphatic hydrocarbons such as oils and waxes (Table 1). No changes have been found in intensities or positions of these bands in the spectra collected from leaves of different glyphosate-resistant plants (Figure 4A) compared with respective non-treated. At the same time, a decrease in the intensity of carotenoids (1156, 1186, and 1525 cm^–1^) has been observed in Raman spectra collected at both D1 and D2 from leaves of susceptible plants after the application of glyphosate, compared with non-treated susceptible plants (Figure 4B).

**FIGURE 5:**
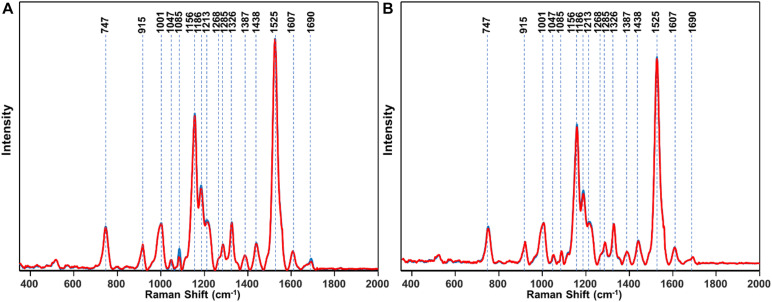
Area-normalized Raman spectra of all resistant (blue) and susceptible (red) populations of Palmer amaranth collected at D1 **(A)** and D2 **(B)**. Here, D1 and D2 indicate observations conducted at 1 and 2 days after herbicide treatment, respectively.

**FIGURE 6:**
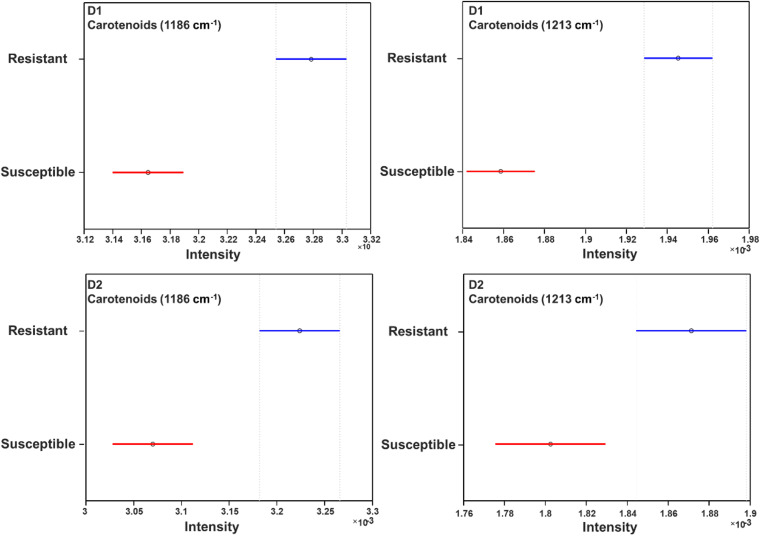
Mean (circles) and 95% confidence intervals for the intensities of weed spectra collected from D1 and D2, normalized to the total spectra area at carotenoid bands 1186 cm^–1^ and 1213 cm^–1^, generated following the ANOVA test. Blue: resistant population, red: susceptible population. D1 = 1 day after treatment and D2 = 2 days after treatment of glyphosate.

About 600 spectra from leaves of three glyphosate-resistant (TX15-14-1, TX15-10, and TX15-12-2) and four -susceptible (TX16-10, TX15-2, TX15-29, and TX15-13-2) populations of Palmer amaranth indicated spectroscopic differences between the two groups following glyphosate treatment. A decrease in the intensities of carotenoid vibrations (1186 and 1213 cm^–1^) has been observed in the spectra of susceptible plants after herbicide application at both D1 and D2 (Figures 5A,B, 6). These results showed consistent reduction in the intensities of carotenoids across different susceptible populations of Palmer amaranth. Two PLS-DA models built for D1 and D2 distinguished the resistant and susceptible populations (Table 4).

**TABLE 4:** PLS-DA confusion matrix of three different resistant and four different susceptible populations.

	Members	True prediction rate (TPR%)	Predicted as non-treated	Predicted as treated
One day after treatment (D1)				
Resistant	353	84.7	299	54
Susceptible	128	71.9	36	92
Matthew’s correlation coefficient (D1) = 0.544				
Two days after treatment (D2)				
Resistant	342	79.8	273	69
Susceptible	104	58.7	43	61
Matthew’s correlation coefficient (D2)^a^ = 0.358				

*^a^Matthew’s correlation coefficient is generated by MATLAB R2019a add-on PLS_Toolbox 8.6.2 (Eigenvector Research Inc.) for the binary classification model. The score of MCC is based on four confusion matrix categories: true positive, false negative, true negative, and false positive to reflect the quality of prediction model.*

Partial least square discriminant analysis is a classification method evolved from PLS which is commonly used for linear regression. PLS-DA is an ideal algorithm for predictive and descriptive modeling (Lee et al., 2018). PLS-DA has quantitative explanatory and qualitative response variables. In the current study, Raman spectra are the quantitative explanatory variables, which have its own explanatory (Raman Shift) and response (intensity) variables. The qualitative response variables are the treatment statuses of the Palmer amaranth.

Besides, PLS-DA, there are other methods like Fisher’s linear discriminant analysis (LDA) and soft independent modeling of class analogies (SIMCA) to build a classification model. LDA assumes that the data will fit in a particular distribution or linear relationships. It is applicable to many classification problems, but it is limited by the correlation between variables (Lee et al., 2018). PLS-DA, comparing with LDA, offers a higher degree of flexibility. Another commonly used method “SIMCA” differentiate different classes using principal component analysis (PCA).

**FIGURE 7:**
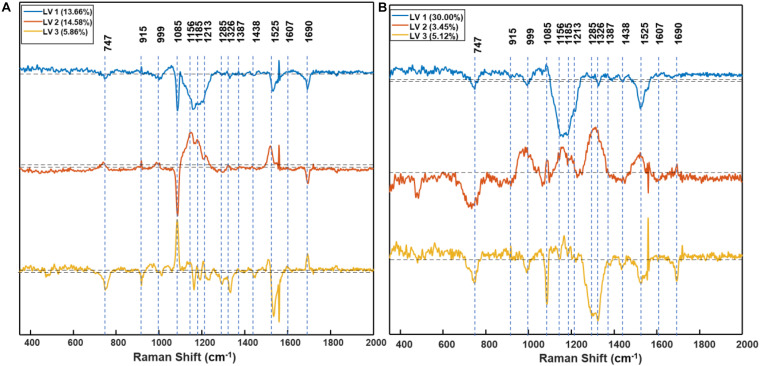
Partial least square discriminant analysis (PLS-DA) model based plot of the first three latent variables (LVs), LV1 (blue), LV2 (orange), and LV3 (yellow) of D1 **(A)** and D2 **(B)** resistant vs. susceptible model. Annotations indicate the centers of the peaks before the first derivative was taken. The dash line in the middle corresponds to 0 point. D1 = 1 day after treatment, and D2 = 2 days after treatment of glyphosate.

Based on PLS-DA models, the first three latent variables of the D1 model (Figure 7A) explained 13.7, 14.6, and 5.9% of the variation between the resistant and susceptible population spectra. The second plot for the latent variables (which explains the greatest class-to-class variation of 14.6%) is similar to the general weed leaf spectrum, suggesting that most of our classifications were made based on the difference in the intensity of the peaks. The carotenoid bands at 1156 to 1213 cm^–1^ and 1525 cm^–1^ expressed greater difference in intensity. A large absolute value at the 1185-cm^–1^ band corresponds to carotenoids. The first three LVs of the D2 model (Figure 7B) explain 30, 3.5, and 5.1% of the variation. In addition to 1156-, 1186-, and 1525-cm^–1^ bands, LV2 and LV3 also express difference at 1285 and 1326 cm^–1^ bands, which are attributed to CH_2_ vibration (Table 1 and Figure 7B). Our results show that glyphosate-resistant and -susceptible populations of Palmer amaranth can be detected with an accuracy of 82.3 and 65.3%, respectively, averaged across D1 and D2. The resistant populations used in this study were 13- to 44-fold resistant (Table 1), which could be easily distinguished from the susceptible populations with consistent accuracies. The relatively lower prediction accuracy of susceptible populations in a general model can be explained by small intra-population variations in their sensitivity to glyphosate. The susceptible populations were highly sensitive to glyphosate at the recommended label rate, and even a small variation in sensitivity could lead to inconsistencies in spectra (Figures 2, 3 and Table 2). Thus, such populations can show a physiological response that can be expressed slightly faster or slower at D1 and D2. Nevertheless, our results show that the prediction accuracy is high enough for confirmatory identification of glyphosate-resistant and -susceptible populations of Palmer amaranth at D1 and D2.

**FIGURE 8:**
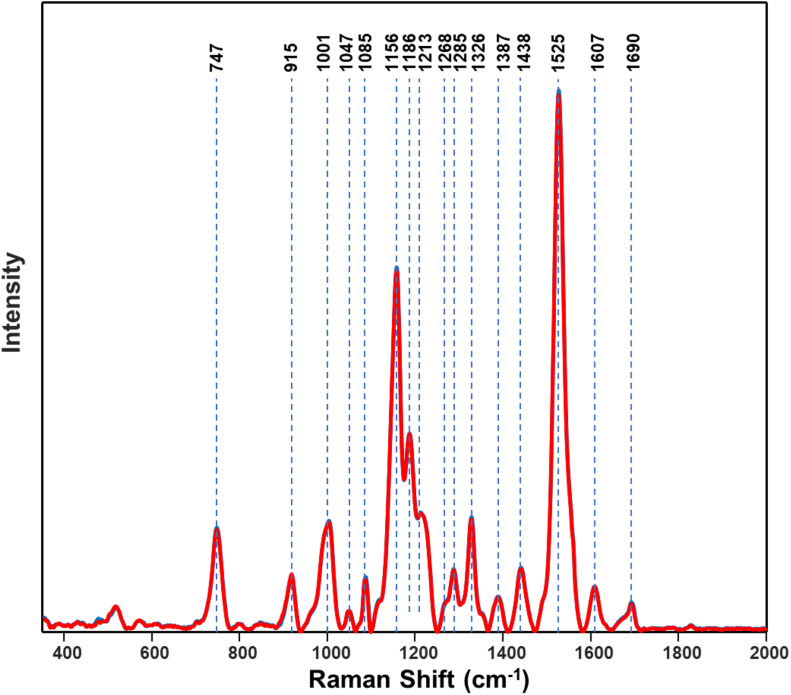
Normalized Raman spectra of resistant (blue) and susceptible (red) populations of Palmer amaranth collected at D0 (non-treated). Spectra of resistant and susceptible at D0 are overlapping.

Spectroscopic signatures of three glyphosate-resistant (TX15-14-1, TX15-10, and TX15-12-2) and four -susceptible (TX15-2, TX15-29, TX15-13-2, and TX16-10) populations of Palmer amaranth were also compared in the absence of herbicide treatment (Figure 8). At D0 (non-treated), spectra from herbicide-resistant populations were not different from the spectra collected from the susceptible Palmer amaranth population. The analysis of variance (ANOVA) test performed at the two carotenoid bands 1186 cm^–1^ and 1213 cm^–1^ showed significant differences at D1 and D2, but not at D0. However, PLS-DA modeling of these spectra allowed for a prediction accuracy of 76.8% for the resistant and 64.8% for the susceptible plants at D0 (Table 5). The PLS-DA analysis may not be picking up direct plant response and needs further research for differentiating herbicide-resistant and -susceptible populations without herbicide applications.

**TABLE 5:** PLS-DA confusion matrix of three non-treated resistant and four non-treated susceptible populations at D0 (before herbicide application).

	Members	True prediction rate (TPR%)	Predicted as resistant	Predicted as susceptible
Resistant	405	76.8	307	98
Susceptible	250	64.8	88	162

*Matthew’s correlation coefficient^a^ = 0.403. ^a^Matthew’s correlation coefficient is generated by MATLAB R2019a add-on PLS_Toolbox 8.6.2 (Eigenvector Research Inc.) for the binary classification model. The score of MCC is based on four confusion matrix categories: true positive, false negative, true negative, and false positive to reflect the quality of prediction model.*

The results of this study indicate that carotenoid content in plants can be used as a simple measure for monitoring herbicide-induced stress. In this study, glyphosate was the specific herbicide tested to compare Raman spectra differences between glyphosate-resistant and -susceptible Palmer amaranth populations, and whether this response can be the same for other herbicides and weed species is yet to be determined. However, exposure to herbicides constitutes a major abiotic stress for plants, and it is known that plant stress can directly influence carotenoid production (Altangerel et al., 2017). Moreover, it is speculated that general stress response can lead to changes in several compounds, which may subsequently alter carotenoid peaks (Dong and Zhao, 2017). Carotenoids are a large group of polyenes that are directly involved in plant stress responses. Based on the acquired Raman spectra, we can conclude that a concentration of carotenoids decreased in the susceptible plants upon glyphosate-induced stress. This suggests that carotenoids were metabolized into abscisic acid, β-ionone, and β-cyclocitrals, molecular analytes that protect the plant against such an abiotic stress (Nambara and Marion-Poll, 2005; Havaux, 2014). At the same time, no significant changes in the concentration of carotenoids in resistant plants were detected.

The use of additive spray for surface-enhanced Raman spectra (SERS) has been suggested to increase the sensitivity of Raman spectra (Yang et al., 2017). In the current study, the SERS could not be implemented in order to avoid any interaction of the additive spray solution with the herbicide molecules. Nevertheless, our experiments were repeated twice with a new set of seedlings each time, and the differentiation of carotenoid peaks for glyphosate-resistant and -susceptible populations was consistent both times, suggesting the robustness of this approach.

The high accuracy of prediction in the current study indicates the potential of RS for use in herbicide-related studies. For example, it can be utilized for detecting herbicide residues in plants and the extent of herbicide drift in crop fields. Herbicide drift is an important field issue in recent times leading to several litigations, and RS may provide an effective means for detecting drift in large field scales. Among the Raman platforms, handheld or remote Raman units are expected to provide more flexibility and convenience for studying plant physiological characteristics from a distance. Recently, a telescopic pulsed Raman system has also been developed (Misra et al., 2005), which is capable of measuring Raman spectra irrespective of light conditions, from a distance of 10 cm to 120 m. This would extend the applications of the Raman system to be utilized in manned or unmanned aerial systems for precision diagnosis of herbicide-related issues under field conditions, which can inform precision weed management.

## Conclusion

The results of this study suggest that RS holds promise for early and rapid field diagnosis of glyphosate-resistant populations and utilization in precision weed management. Raman spectra could differentiate herbicide-treated and non-treated susceptible populations with an accuracy of 93.5 and 82.1%, respectively, averaged across D1 and D2. Based on PLS-DA modeling, the glyphosate-resistant and -susceptible populations of Palmer amaranth can be easily predicted with an accuracy of 84.7 and 71.9%, respectively, at D1. The accuracy of predicting a glyphosate-resistant and -susceptible population without herbicide treatment was 76.8 and 64.8%, respectively, but the differences were statistically non-significant. More research is required to detect subtle differences in Raman spectra to differentiate herbicide-resistant and -susceptible populations prior to herbicide application, although it is unclear if such differences can be case-specific and any generalizations could be made. Standardization of this can bring revolutionary changes in herbicide resistance detection and management approaches. Nevertheless, the availability of hand-held Raman sensors provides opportunities for rapid detection of herbicide resistance and stress response under field conditions. More research is imperative for improving the utility of this technology for broader applications.

## Data Availability Statement

The raw data supporting the conclusions of this article will be made available by the authors, without undue reservation.

## Author Contributions

MB, VS, and DK conceptualized the study and interpreted the results. VS, DV, and AP performed the preliminary studies. SS conducted the herbicide assays. VS and SS conducted molecular studies for estimating *EPSPS* gene copy numbers. TD, MK, DH, WP, and LS have performed Raman measurements reported in the manuscript. TD performed the statistical analysis of data. All authors reviewed the manuscript.

## Conflict of Interest

The authors declare that the research was conducted in the absence of any commercial or financial relationships that could be construed as a potential conflict of interest.

## Supplementary Material

Supplementary Figure 1 Raman spectra of resistant (blue) and susceptible (red) populations of Palmer amaranth collected at D1 **(A)** and D2 **(B)**, where D1 = 1 day after treatment and D2 = 2 days after treatment of glyphosate.

Supplementary Figure 2 Mean (circles) and 95% confidence intervals for the intensities of Raman spectra collected D0 weed spectra, normalized to the total spectral area, at carotenoid bands **(A)** 1186 cm^–1^ and **(B)** 1213 cm^–1^, generated following the ANOVA test. Blue: resistant population, gray: susceptible population. Here, D0 is before herbicide application (non-treated).
