# Supplementary figures and images for: Raman Spectroscopy Can Distinguish Glyphosate-Susceptible and -Resistant Palmer Amaranth (Amaranthus palmeri)
Source: Front Plant Sci. 2021 Jun 4;12:657963. doi: 10.3389/fpls.2021.657963 (PMC8212978; doi:10.3389/fpls.2021.657963)

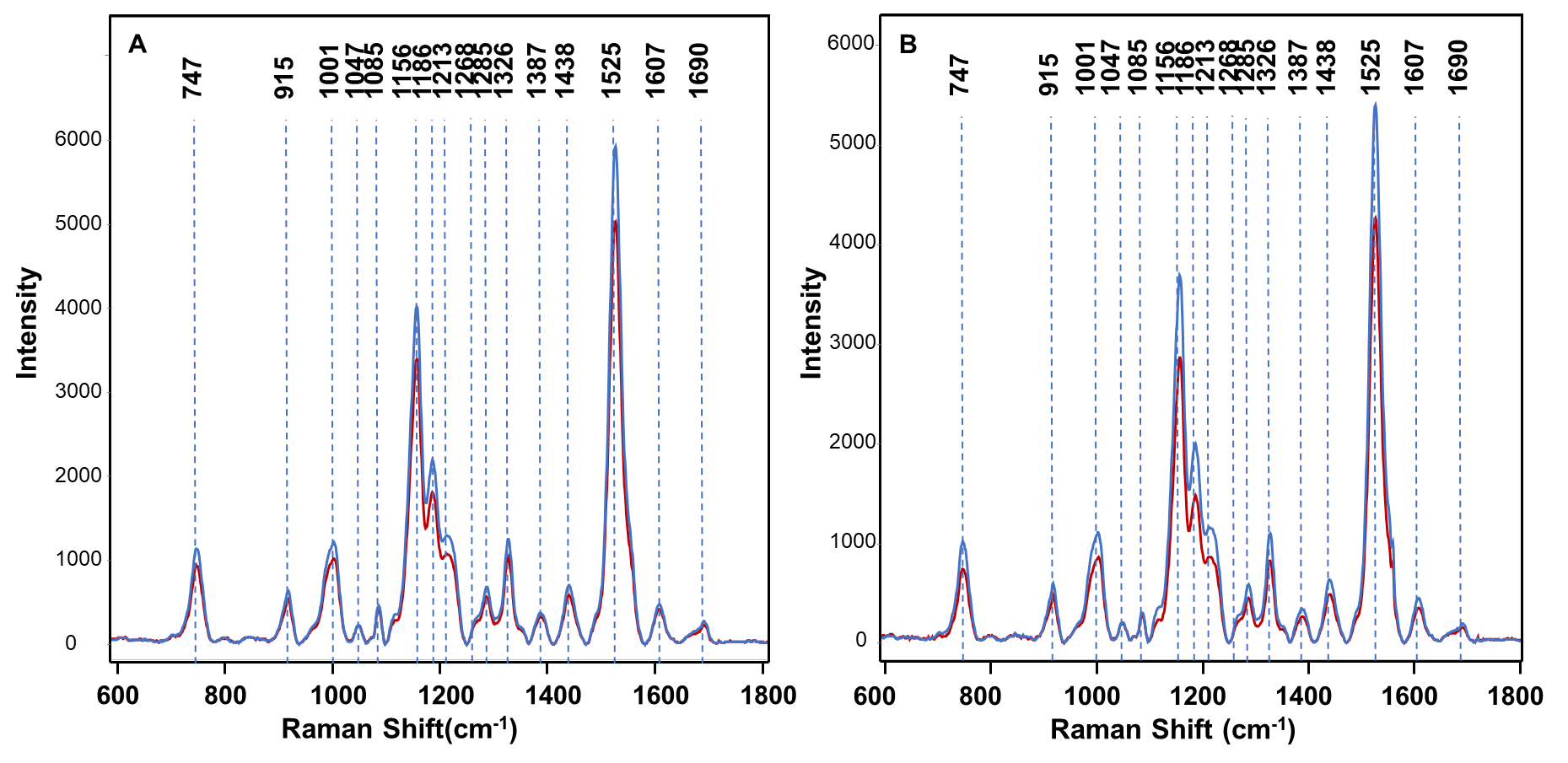

Supplement: Supplementary Figure 1 — Raman spectra of resistant (blue) and susceptible (red) populations of Palmer amaranth collected at D1 (A) and D2 (B), where D1 = 1 day after treatment and D2 = 2 days after treatment of glyphosate. [file Image_1.png]

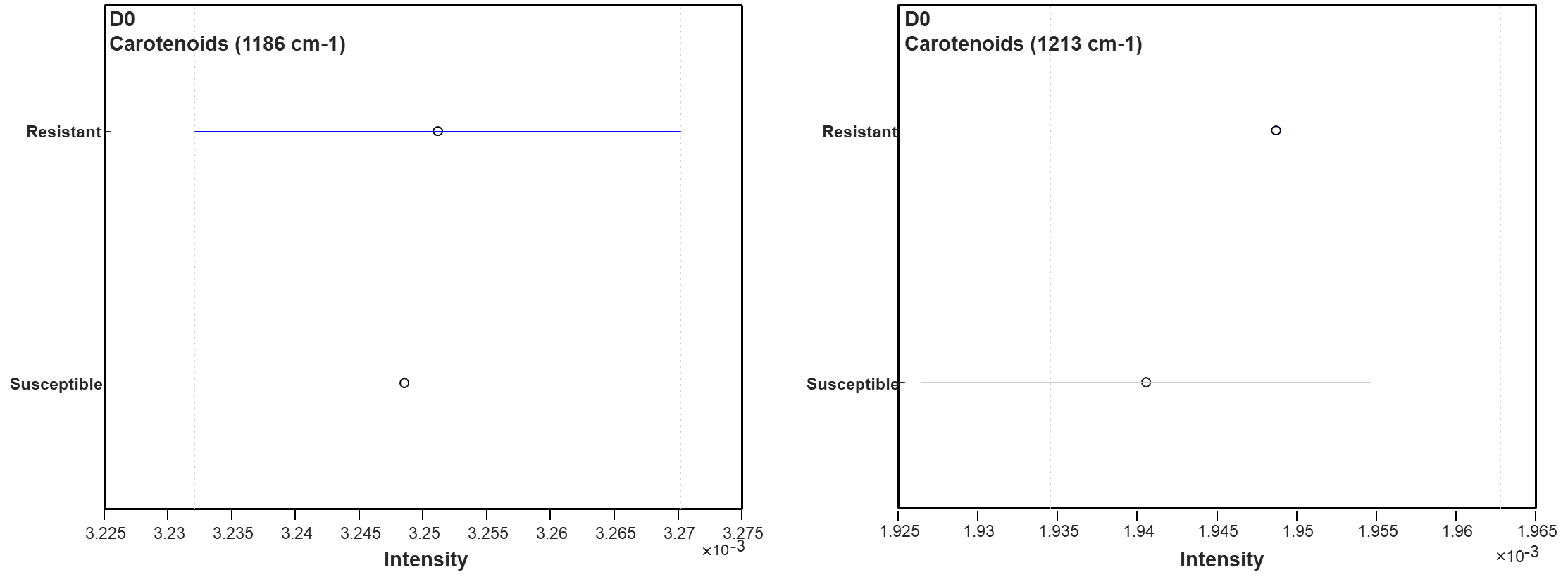

Supplement: Supplementary Figure 2 — Mean (circles) and 95% confidence intervals for the intensities of Raman spectra collected D0 weed spectra, normalized to the total spectral area, at carotenoid bands (A) 1186 cm–1 and (B) 1213 cm–1, generated following the ANOVA test. Blue: resistant population, gray: susceptible population. Here, D0 is before herbicide application (non-treated). [file Image_2.png]
